# Blue light‐emitting diodes induce autophagy in colon cancer cells by Opsin 3

**DOI:** 10.1002/ags3.12055

**Published:** 2018-01-11

**Authors:** Toshiaki Yoshimoto, Yuji Morine, Chie Takasu, Rui Feng, Tetsuya Ikemoto, Kozo Yoshikawa, Syuichi Iwahashi, Yu Saito, Hideya Kashihara, Masatake Akutagawa, Takahiro Emoto, Yosuke Kinouchi, Mitsuo Shimada

**Affiliations:** ^1^ Department of Surgery Tokushima University Graduate School Tokushima Japan; ^2^ Graduate School of Technology, Industrial and Social Sciences Tokushima University Tokushima Japan; ^3^ Center of Research Administration & Collaboration Tokushima University Tokushima Japan

**Keywords:** autophagy, blue light‐emitting diode, colorectal cancer, Opsin 3, photoreceptor

## Abstract

**Background:**

Light emitting‐diodes (LED) have various effects on living organisms and recent studies have shown the efficacy of visible light irradiation from LED for anticancer therapies. However, the mechanism of LED's effects on cancer cells remains unclear. The aim of the present study was to investigate the effects of LED on colon cancer cell lines and the role of photoreceptor Opsin 3 (Opn3) on LED irradiation in vitro.

**Methods:**

Human colon cancer cells (HT‐29 or HCT‐116) were seeded onto laboratory dishes and irradiated with 465‐nm LED at 30 mW/cm^2^ for 30 minutes. Cell Counting Kit‐8 was used to measure cell viability, and apoptosis and caspase 3/8 expression were evaluated by AnnexinV/PI and reverse transcription‐polymerase chain reaction (RT‐PCR), respectively. Autophagy and expression of LC‐3 and beclin‐1 were also evaluated by autophagy assays, RT‐PCR and Western blotting. We further tested Opn3 knockdown by Opn3 siRNA and the G_i/o_ G‐protein inhibitor NF023 in these assays.

**Results:**

Viability of HT‐29 and HCT‐116 cells was lower in 465‐nm LED‐irradiated cultures than in control cultures. LC‐3 and beclin‐1 expressions were significantly higher in LED‐irradiated cultures, and autophagosomes were detected in irradiated cells. The reductive effect of cancer cell viability following blue LED irradiation was reversed by Opn3 knockdown or NF023 treatment. Furthermore, increased LC‐3 and beclin‐1 expression that resulted from blue LED irradiation was suppressed by Opn3 knockdown or NF023 treatment.

**Conclusion:**

Blue LED irradiation suppressed the growth of colon cancer cells and Opn3 may play an important role as a photoreceptor.

## 1 INTRODUCTION

Light‐emitting diodes (LED) are the preferred light source for phototherapy and have been developed to replace traditional light bulbs because of their lower cost and high variability of wavelengths.^1^ LED have been used to treat dermatitis^2^ and muscle analgesia,^3^ as well as to remove bacteria in vitro.^4^ Recent studies have reported that blue light irradiation inhibits cell growth in various cancers in vitro and in vivo.^1^, ^5^, ^6^, ^7^ Phototoxic and antiproliferative mechanisms of blue light LED include inducing apoptosis,^6^ autophagy^1^ and cell cycle arrest.^7^ We also reported that blue LED irradiation at 465 nm inhibited proliferation of HT‐29 and HCT‐116 cells by extrinsic apoptosis and mitogen‐activated protein kinase (MAPK) pathways.^8^ However, the mechanism of light reception in these cells, especially “non‐visual” tumor cells, is unclear.

In animals, opsin‐based photopigments act as photoreceptors for vision and other non‐visual functions, such as circadian photoentrainment and pupil response.^9^ Most animal opsin‐based pigments are typical G protein‐coupled receptors (GPCR).^10^ Based on their amino acid sequences, opsins are currently classified into seven distinct groups, and more than 1000 opsin subtypes have been found in vertebrates.^11^ Within those subtypes, Opsin 3 (Opn3), originally called encephalopsin, panopsin or teleost multiple tissue opsin, was first identified in the deep brain and internal organs of humans and mice.^10^ Opn3 exhibits an absorption maxima at 460‐470 nm and activation efficiencies of the G_i_ and G_o_ subtypes of G protein, which inhibits adenylate cyclase and decreases cyclic adenosine monophosphate (cAMP).^12^, ^13^ Interestingly, Opn3 and its homologs are expressed in various non‐visual tissues, such as the brain, liver, kidney and heart; furthermore, they work as photoreceptors and regulate GPCR signaling.^12^ A previous report showed that opsins relate to phototherapy as photoreceptors in malignant melanocytes;^14^ however, the role of Opn3 in colon cancer is unclear.

The aim of the present study was to investigate the effects of blue LED irradiation on colon cancer cells and to further dissect whether Opn3 functions as a photoreceptor in this process.

## 2 MATERIALS AND METHODS

### 2.1 Cell cultures and reagents

Human colon cancer cell lines HT‐29 and HCT‐116 were purchased from the American Type Culture Collection (ATCC, Manassas, VA, USA). Cells were maintained in RPMI 1640 medium (WAKO, Osaka, Japan) containing 10% fetal bovine serum, 100 U/mL penicillin and 100 μg/mL streptomycin (Sigma‐Aldrich, St Louis, MO, USA) at 37°C in an environment containing 5% CO_2_. NF023 (selective inhibitor of the G_i/o_ α‐subunit of G‐protein) was purchased from Merck Millipore (Darmstadt, Germany).

### 2.2 siRNA transfection

Cells were transfected with Opn3 siRNA (s24172) using Lipofectamine RNAiMAX (Invitrogen/Thermo Fisher Scientific, Waltham, MA, USA). Transfections were carried out at 37°C in a humidified incubator with 5% CO_2_. HT‐29 or HCT‐116 cells were seeded into 35‐mm dishes and transfected with 5 μL siRNA with 5 μL RNAiMAX in a final volume of 2 mL RPMI 1640.

### 2.3 Light‐emitting diodes irradiation

A 465‐nm blue light LED (NCSB119) was used for irradiation experiments (NICHIA Corporation, Tokushima, Japan). A photo‐radiometer (MCPD‐370A; Otsuka Electronics, Tokyo, Japan) was used to measure light intensity. An LED irradiation device (Department of Electrical and Electronic Engineering, Faculty of Engineering, The University of Tokushima, Tokushima, Japan) was used as a platform for the light source. We used the continuous mode and a distance of 15 cm from the light source to the cells for each irradiation; irradiance was set at 30 mW/cm^2^ (1.0 A). For all experiments, 5 × 10^4^ cells/well were seeded with medium onto 35‐mm plates (BD Biosciences, Franklin Lakes, NJ, USA). Cells in the LED treatment group were exposed to 465‐nm wavelength light for 30 minutes in a dark room. For Opn3 knockdown studies, Opn3 siRNA‐transfected cells were exposed to 465 nm wavelength light for 30 minutes 24 hours after transfection. For G_i/o_ G‐protein inhibitor studies, NF023 was used at 50 μmol L^−1^, 24 hours after seeding, and NF023‐administered cells were exposed to 465 nm wavelength light for 30 minutes after 24 hours NF023 treatment. Control cells were treated in the same way except for light exposure. No treatments increased culture medium temperature.

### 2.4 Cell proliferation assay

Cell Counting Kit‐8 (CCK8; Dojindo, Kumamoto, Japan) was used to measure cell proliferation. Briefly, 48 hours after LED irradiation, 200 μL CCK8 was added to each plate. After 2 hours incubation, optical density was biochromatically measured with an enzyme calibrator at 450 and 630 nm.

### 2.5 Apoptosis detection by flow cytometry

Irradiated and control cells were labeled with both Annexin V‐fluorescein isothiocyanate (FITC) and propidium iodide (PI) (eBioscience/Thermo Fisher Scientific). Unexposed cells without Annexin V or PI were used as negative controls. A FACSCalibur flow cytometer (BD Biosciences) was used to measure the proportion of apoptotic cells in each culture at 24 hours after LED irradiation. Quantitative analysis was carried out with WinMDI 2.8 software (The Scripps Institute, San Diego, CA, USA).

### 2.6 RT‐PCR analysis

RNeasy Mini Kit (QIAGEN, Hilden, Germany) was used according to the manufacturer's instructions to prepare each total RNA sample, which was reverse transcribed with a high‐capacity cDNA reverse transcription kit (Applied Biosystems, Tokyo, Japan). The 7500 real‐time PCR system, TaqMan gene expression assays on demand and TaqMan universal master mix (Applied Biosystems) were used to carry out quantitative real‐time PCR. The following TaqMan assays (assay identification number) were used: caspase 3 (Hs00263337_m1), caspase 8 (Hs00236278_m1), LC‐3 (Hs01076567_g1) and beclin‐1 (Hs01061917_g1). GAPDH (4326317E) was used as an internal control for mRNA expression (all from Applied Biosystems). Thermal cycler conditions were as follows: 2 minutes at 50°C, 10 minutes at 95°C, and then 40 cycles of 15 seconds at 95°C and 1 minute at 60°C. Amplification data were analyzed with the Prism 7500 Sequence Detection System ver. 1.3.1 (Applied Biosystems).

### 2.7 Detection of autophagosomes

Autophagosomes were detected with a Cell Meter Autophagy kit and Autophagy Blue™ (AAT Bioquest, Inc., Sunnyvale, CA, USA) at 24 hours after LED irradiation. Irradiated and control cells were stained with an Autophagy Blue™ working solution 48 hours after LED irradiation and incubated at 37°C for 1 hour. Cells were washed three times and examined under a fluorescence microscope (Ex/Em = 335/520 nm).

#### 2.7.1 Western blotting

Cells were harvested and lysed using RIPA buffer (Thermo Fisher Scientific) in the presence of the protease inhibitor cocktail (Sigma‐Aldrich) at 24 hours after LED irradiation. Proteins were then quantified with a BCA kit (PA115; Tiangen Biotech, Beijing, China), resolved on 10% sodium dodecyl sulfate‐polyacrylamide gel electrophoresis (SDS‐PAGE) gels, and transferred onto polyvinylidene difluoride (PVDF) membranes (162‐0177; Bio‐Rad, Hercules, CA, USA). Membranes were incubated with the appropriate primary antibody followed by incubation with the corresponding horseradish peroxidase (HRP)‐conjugated secondary antibody, and proteins were detected with enhanced chemiluminescence (ECL) Western blotting substrates (W1001; Promega, Madison, WI, USA).

#### 2.7.2 Opsin 3 expression analysis by immunohistochemistry

This study was carried out in accordance with the Helsinki Declaration of the World Medical Association. Retrospectively, tumor samples of colon cancer were collected from formalin‐fixed paraffin‐embedded specimens that were resected at surgical operation in our institute. Slide was immunostained using Anti‐Opn3 antibody (SAB2700986; Sigma‐Aldrich) according to the manufacturer's instructions.

### 2.8 Statistical analysis

All statistical analyses were done using Stat View version 5.0 (SAS Institute, Cary, NC, USA). Mann‐Whitney *U*‐test and the Wilcoxon signed‐rank test were used for statistical comparisons. Differences were considered statistically significant at *P < *.05.

## 3 RESULTS

### 3.1 Effects of blue LED irradiation on cell proliferation

**Figure 1:**
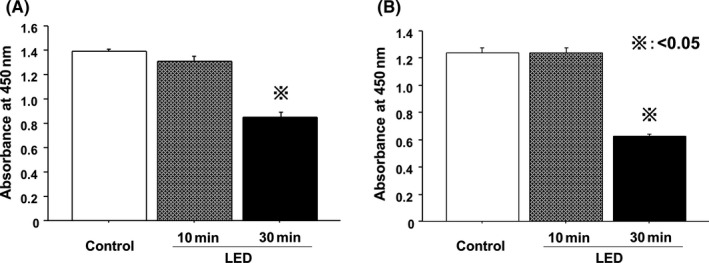
Effect of blue light‐emitting diodes (LED) on cell viability. Viability of (A) HT‐29 and (B) HCT‐116 cells exposed to blue LED for 30 minutes was significantly reduced

Light‐emitting diodes emitting 465 nm light were used to assess the effects of blue LED irradiation on HT‐29 and HCT‐116 cell proliferation. Cells were irradiated once for 30 minutes, and then CCK‐8 assays were carried out 48 hours after irradiation to measure cell viability. Viability of HT‐29 and HCT‐116 cells exposed to blue LED was significantly reduced (Figure 1).

### 3.2 Effects of blue LED irradiation on apoptosis

**Figure 2:**
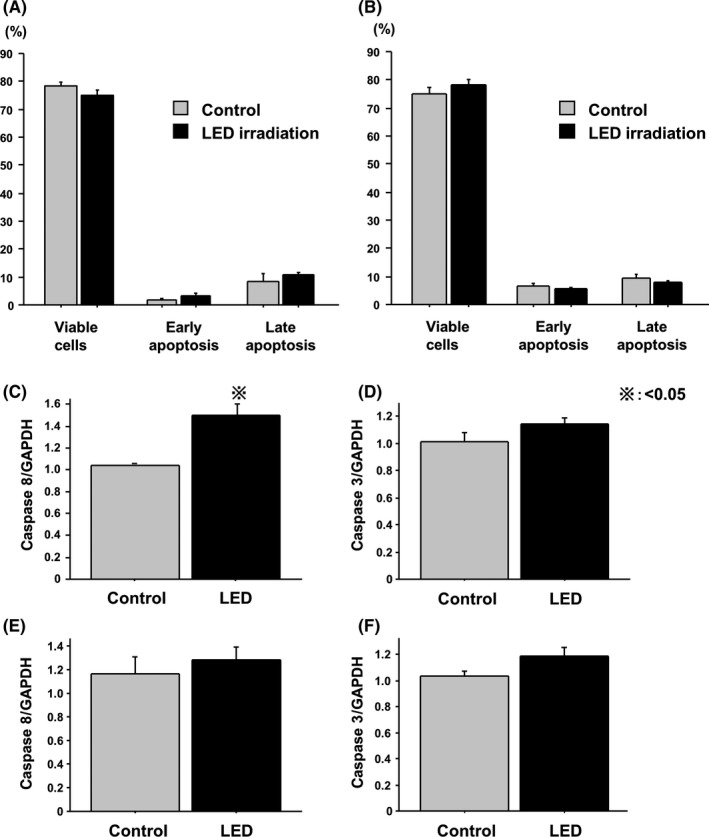
Effect of blue light‐emitting diodes on apoptosis. Apoptosis was not detected in (A) HT‐29 and (B) HCT‐116 cells. (C) Caspase 8 expression was significantly higher in irradiated HT‐29 cells compared with control cells. (D‐F) Caspase 8 in HCT‐116 cells and caspase 3 in both cell lines did not show significant changes

Annexin V flow cytometry experiments were carried out to assess whether 465‐nm blue LED irradiation led to increased apoptosis in HT‐29 and HCT‐116 cells. Results showed that apoptosis was not induced in HT‐29 and HCT‐116 cells (Figure 2A,B). Expression of two pro‐apoptotic factors (caspase 8 and 3) was then analyzed by RT‐PCR at 24 hours after LED irradiation. Caspase 8 expression was significantly higher in irradiated HT‐29 cells compared with control cells (Figure 2C). However, caspase 8 in HCT‐116 cells and caspase 3 in both cell lines did not show significant changes (Figure 2D‐F).

### 3.3 Blue LED irradiation induces autophagy in HT‐29 and HCT‐116 cells

**Figure 3:**
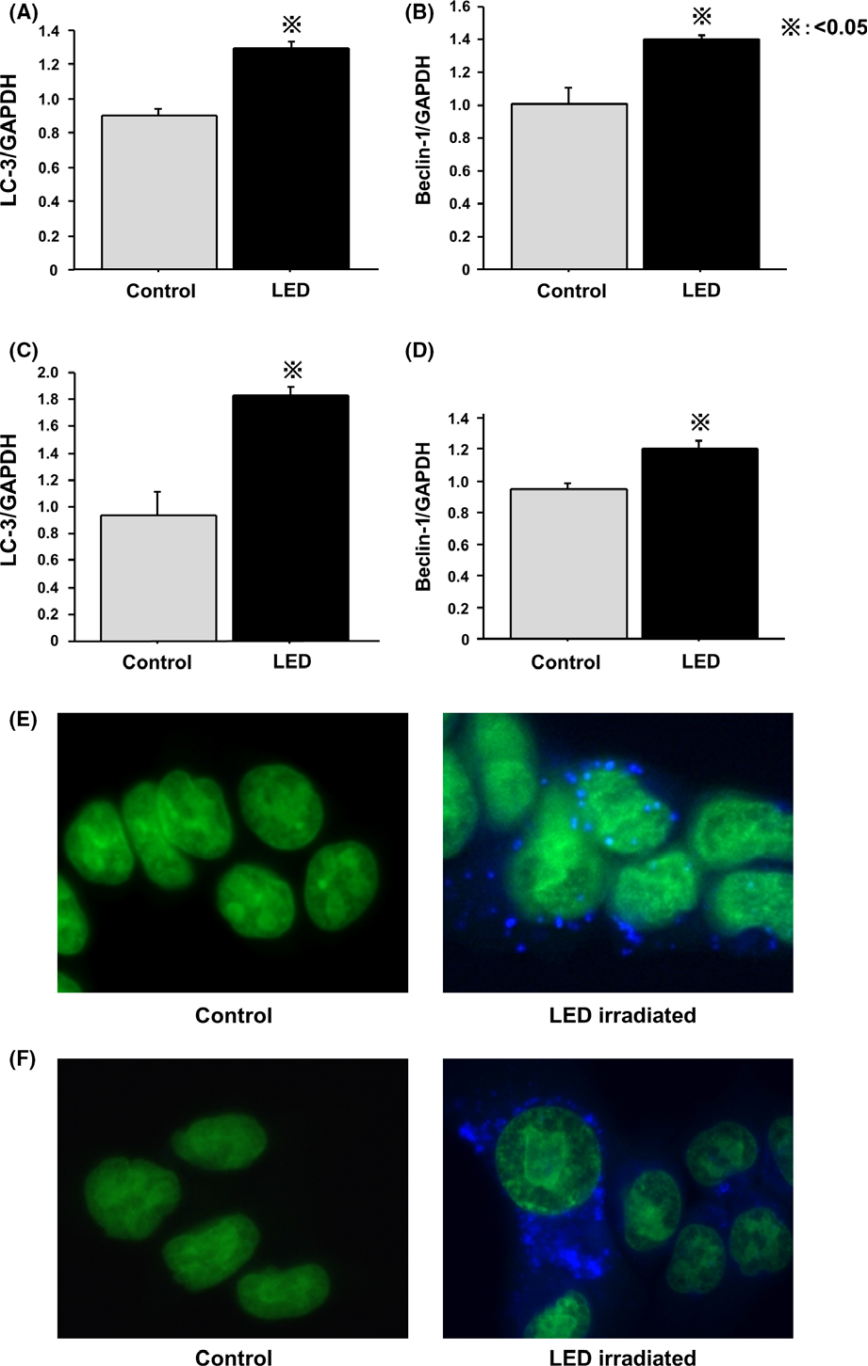
Blue light‐emitting diodes exposure induces autophagy in colon cancer cells lines. LC‐3 and beclin‐1 mRNA levels were significantly higher in irradiated (A,B) HT‐29 and (C,D) HCT‐116 than in control cells. Autophagosomes were detected in irradiated (E) HT‐29 and (F) HCT‐116

Expressions of autophagy‐related genes were analyzed by RT‐PCR at 24 hours after LED irradiation. LC‐3 and beclin‐1 expression were significantly higher in irradiated HT‐29 (Figure 3A,B) and HCT‐116 (Figure 3C,D) than in control cells. Moreover, autophagosomes were detected in irradiated HT‐29 (Figure 3E) and HCT‐116 cells (Figure 3F).

### 3.4 Role of Opn3 on the effects of blue light irradiation

**Figure 4:**
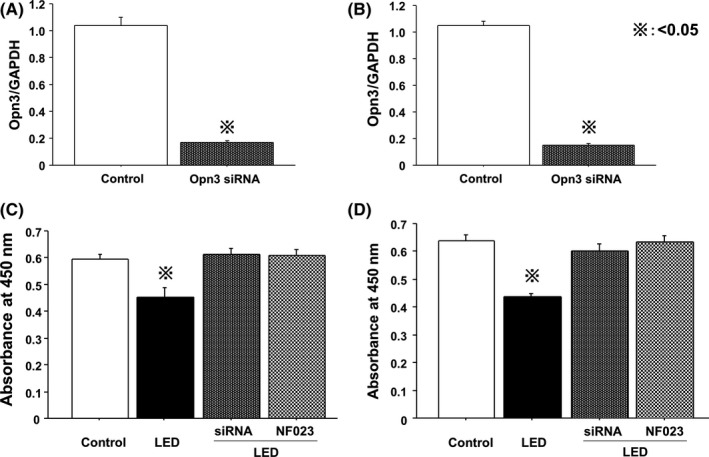
Opsin 3 (Opn3) knockdown or NF023 treatment reverses the cytotoxic effect of blue light‐emitting diodes (LED). Opn3 was silenced in (A) HT‐29 and (B) HCT‐116 cells by Opn3 siRNA. Viability of Opn3 knockdown cells with LED irradiation and cells treated with NF023 and LED were significantly increased compared with the LED irradiation‐only group in (C) HT‐29 and (D) HCT‐116

**Figure 5:**
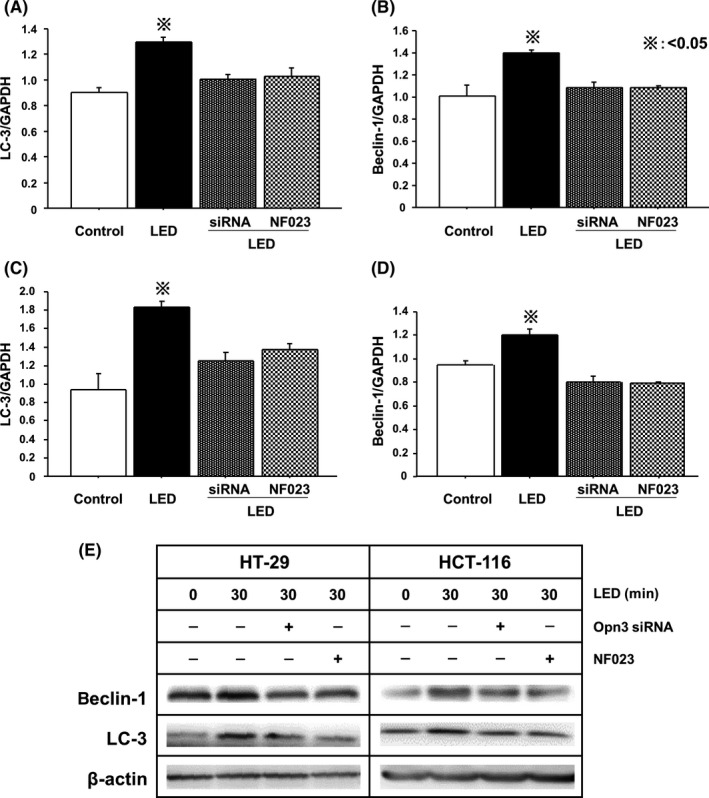
Opsin 3 (Opn3) knockdown or NF023 inhibits the upregulation of LC‐3 and beclin‐1 caused by blue light‐emitting diodes (LED). Upregulation of LC‐3 and beclin‐1 mRNA expressions by blue LED were suppressed by Opn3 knockdown or NF023 treatment compared with the LED irradiation‐only group in (A,B) HT‐29 and (C,D) HCT‐116. Protein expressions of LC‐3 and beclin‐1 in the above conditions were determined by (E) Western blotting

We next knocked down Opn3 in HT‐29 and HCT‐116 cells using Opn3 siRNA (Figure 4A,B). Viabilities of both Opn3 knockdown cells with blue LED and cells treated with NF023 and blue LED were significantly increased compared with the blue LED‐only controls (Figure 4C,D). Increased LC‐3 and beclin‐1 mRNA expression following blue LED irradiation was significantly suppressed in Opn3 knockdown cells and in cells treated with NF023 (Figure 5A‐D).

To examine protein expressions of these autophagy‐related proteins, Western blotting was carried out. In HT‐29 and HCT‐116 cells, expressions of LC‐3 and beclin‐1 were increased by blue LED irradiation, compared to control cells. In contrast, these upregulations of LC‐3 and beclin‐1 were suppressed in Opn3 knockdown cells and in cells treated with NF023 (Figure 5E).

#### 3.4.1 Opn3 protein expression in human colon cancer tissue

**Figure 6:**
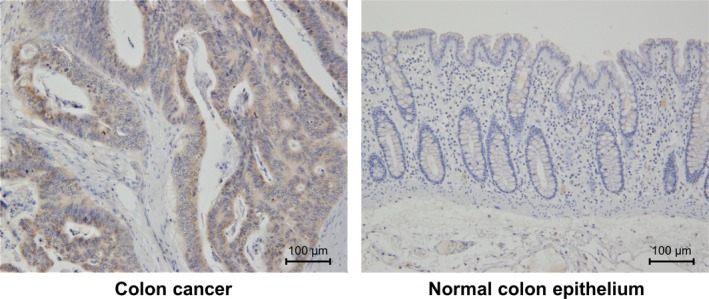
Opsin 3 (Opn3) expression by immunohistochemistry in human colon cancer tissue. Human colon cancer cells were positive for Opn3 and normal colon epithelial cells showed weak positive (magnification × 200)

To investigate protein expression of Opn3 in human colon cancer, immunohistochemistry staining was carried out in tissue resected by surgical operation in our institute. In our case, Opn3 expression was observed in human colon cancer cells and normal colon tissue was weakly positive for Opn3 (Figure 6).

## 4 DISCUSSION

This study showed that 30 minutes irradiation with 465‐nm blue LED inhibited the growth of human colon cancer cell lines, and blue LED‐induced autophagy was shown to be the mechanism of cell growth inhibition. Additionally, the effects of LED were inhibited by Opn3 knockdown or selective inhibition of the G_i/o_ G‐protein α‐subunit. These results suggested that irradiation with blue LED inhibited the growth of colon cancer cells by inducing autophagy through the Opn3 photoreceptor pathway. In addition, immunohistochemistry showed that Opn3 protein was highly expressed in tumor tissue of colon cancer compared to non‐tumor tissue. This finding suggested that blue light irradiation could be a possible clinical application in treating colon cancer.

Blue LED have been reported to have phototoxic and antiproliferative effects through the generation of intracellular reactive oxygen species (ROS)^15^, ^16^ blue LED irradiation has also been reported to induce apoptosis by a mitochondrially mediated signaling pathway that reduces early‐stage melanoma growth.^6^ Recently, we reported that 10 min/d × 5 d of blue LED irradiation induced apoptosis in colon cancer cells.^8^ In this study, the irradiation method was changed to 30 minutes once, and cell growth was inhibited to the same degree; however, there was not a significant induction of apoptosis. Interestingly, we found that autophagy markers were upregulated and autophagosomes were detected in irradiated cells. This exciting result suggested that the inhibitory effect on cell growth in cancer cells differed according to the blue LED irradiation protocol.

Autophagy is one of the catabolic cellular responses that sustains cellular metabolism against starvation or stress, whereby cellular proteins and organelles are engulfed, digested and recycled.^17^, ^18^ Significant evidence supports a role for autophagy in sustaining cell survival, but, paradoxically, progressive cellular consumption by unrestrained autophagy results in cell death.^19^, ^20^ In cancer cells, autophagy may induce caspase‐independent cell death and suppress tumor progression.^19^ Previous reports have shown that autophagy is negatively regulated by cAMP signaling.^21^, ^22^ Furthermore, Oh et al^1^ showed that blue LED induced autophagy and reduced cell viability in lymphoid cells. Several mechanisms of the cytotoxic effect of blue LED have been reported, and irradiation time‐dependent effects have been suggested, such as in melanoma cells, in which cell cycle arrest increased in a time‐dependent way.^6^ In regard to autophagy, longer irradiation time with blue LED increased LC‐3 expression and induced the formation of autophagosomes.^1^ Our previous report showed induction of apoptosis by short daily blue LED irradiation times; however, this study showed that autophagy was induced after a single irradiation of longer duration. The relationship between apoptosis and autophagy is rather complicated,^8^ as autophagy can either delay or promote apoptosis.^23^, ^24^ This study showed that blue LED irradiation has time‐dependent effects on colon cancer cells.

Several reports have shown the cytotoxic effect of blue LED, but the cellular mechanism of light reception remained unclear. A wide variety of opsins exist in vertebrates as photoreceptors, and each has an optimal wavelength. The first report of the relationship between opsins and tumor suppression was reported by Yang et al,^25^ who showed that opsin gene transfers with blue LED irradiation inhibited the growth of malignant glioma. In the opsin family, Opn3 is known as a blue light receptor and is widely expressed in non‐visual cells. In this study, human colon cancer cell lines also expressed Opn3 and, from the fact that blue LED's effect was inhibited by Opn3 knockdown or inhibiting the G_i/o_ G‐protein α‐subunit, Opn3 may play an important role as a G protein‐coupled photoreceptor by cAMP signaling in inducing autophagy.

Based on these results, we conclude that blue LED irradiation induces autophagy and suppresses the growth of colon cancer cells by the Opn3 photoreceptor pathway, which regulates the G_i/o_ G‐protein α‐subunit. Although further investigation is required to fully elucidate the molecular mechanism of blue LED irradiation, including phosphorylation, G‐protein function, cAMP signaling and their relationship with apoptosis, this study showed a novel mechanism of blue LED irradiation by Opn3 and we consider that Opn3 could be an important target of colon cancer in clinical settings. In the future, there is the possibility of the development of phototherapy for colon cancer by irradiation from the tip of an endoscope for example.

## DISCLOSURE

Conflicts of Interest: Authors declare no conflicts of interest for this article.
